# A decision support system for assessing management interventions in a mental health ecosystem: The case of Bizkaia (Basque Country, Spain)

**DOI:** 10.1371/journal.pone.0212179

**Published:** 2019-02-14

**Authors:** Carlos R. García-Alonso, Nerea Almeda, José A. Salinas-Pérez, Mencía R. Gutiérrez-Colosía, José J. Uriarte-Uriarte, Luis Salvador-Carulla

**Affiliations:** 1 Universidad Loyola Andalucía, Seville, Spain; 2 Bizkaia Mental Health Services, Osakidetza-Basque Health Service, Biocruces Health Research Institute, Bilbao, Spain; 3 ANU College of Health and Medicine, Australian National University, Canberra, Australia; University of Murcia, SPAIN

## Abstract

Evidence-informed strategic planning is a top priority in Mental Health (MH) due to the burden associated with this group of disorders and its societal costs. However, MH systems are highly complex, and decision support tools should follow a systems thinking approach that incorporates expert knowledge. The aim of this paper is to introduce a new Decision Support System (DSS) to improve knowledge on the health ecosystem, resource allocation and management in regional MH planning. The Efficient Decision Support-Mental Health (EDeS-MH) is a DSS that integrates an operational model to assess the Relative Technical Efficiency (RTE) of small health areas, a Monte-Carlo simulation engine (that carries out the Monte-Carlo simulation technique), a fuzzy inference engine prototype and basic statistics as well as system stability and entropy indicators. The stability indicator assesses the sensitivity of the model results due to data variations (derived from structural changes). The entropy indicator assesses the inner uncertainty of the results. RTE is multidimensional, that is, it was evaluated by using 15 variable combinations called scenarios. Each scenario, designed by experts in MH planning, has its own meaning based on different types of care. Three management interventions on the MH system in Bizkaia were analysed using key performance indicators of the service availability, placement capacity in day care, health care workforce capacity, and resource utilisation data of hospital and community care. The potential impact of these interventions has been assessed at both local and system levels. The system reacts positively to the proposals by a slight increase in its efficiency and stability (and its corresponding decrease in the entropy). However, depending on the analysed scenario, RTE, stability and entropy statistics can have a positive, neutral or negative behaviour. Using this information, decision makers can design new specific interventions/policies. EDeS-MH has been tested and face-validated in a real management situation in the Bizkaia MH system.

## Introduction

Decision Analysis (DA) is a technique to support decision making avoiding pitfalls. DA aims to understand the decision-making processes, the factors involved in the way people make decisions and the procedures involved in choosing alternatives for maximizing the expected utility, the probability of achieving some specific goals or minimizing the decisional uncertainty [1].

The recommendations proposed by the World Health Organization (2018) [2] for Mental Health (MH) policy development highlight the need to help policy makers to reach deeper knowledge on service planning, but traditional approaches focusing on systematic reviews of evidence, policy briefs and better accessibility to data [3] do not suffice to guide decision making. In real world conditions, decision makers might approximate the consequences of a policy plan or specific management interventions (e.g., reallocation of workforce capacity or beds) in a very limited way. Additionally, the complexity, uncertainty, non-linearity, dimensionality and multiscalarity of the questions posed in mental healthcare planning [4] make it necessary to integrate an entire array of different disciplines, research fields and analysis techniques to develop usable and interoperable Decision Support Systems (DSS) in the real world.

DSS are interactive computer-based tools for improving decision-making processes and guiding decision makers in semi-structured or, sometimes, unstructured problem solving [5,6]. DSS integrate techniques from: statistics, clinical information systems, computational modelling, simulation and machine learning, among others [7]. Nevertheless, the capacitation processes for guiding users to make decisions based on DSS results are always complex in dynamic environments [8,9].

DSS based on artificial intelligence techniques [10], Bayesian networks [11] and simulation modelling [12] have been applied for decision-making in health care, in general, and in MH care, in particular, [13–15]. Finally, DSS can assess the impact and/or effectiveness of MH policies [16], improving both the management of MH services [17–19] and care provision [20].

The Efficient Decision Support—Mental Health (EDeS-MH) is a DSS based on Relative Technical Efficiency (RTE), Monte-Carlo simulation and artificial intelligence (a fuzzy inference engine prototype for interpreting standard “IF … Then” production rules) that has been developed following the systems thinking approach requirements.

RTE analyses the relationship that exists between the inputs (usually resources) consumed and the outputs (resource utilisation and outcomes) produced by a set of comparable Decision Making Units (DMUs) [21]. These DMUs interact in complex systems, such as the MH ones, in uncertain environments. The RTE is “relative” because it is obtained by comparing every DMU to each other. The DMU that shows the best input/output rates has a RTE = 1 (best efficiency), while the others have RTE scores between a [0, 1) range where an RTE = 0 means a complete inefficiency. The RTE techniques can analyse Small Health Areas (SHAs) (meso-level) as well as the entire MH systems, considering both the real (initial) situation “A” and the consequential potential situation “B” after acting the designed interventions.

Due to the environmental and the inner MH systems’ uncertainties, RTE is probabilistic. Recent findings have evidenced that stochastic RTE is a good indicator to assess the performance of MH services [22].

Regarding to the methodology used for analysing RTE, Data Envelopment Analysis (DEA) is a robust non-parametric technique for assessing performance of a set of comparable DMUs, introduced by Charnes, Cooper and Rhodes in 1978. Till now, DEA has been widely applied in many research fields [23–25] including healthcare [26–28]. Nevertheless, the RTE assessment of the performance of MH services is still a challenge [22,29–31].

In order to solve main drawbacks of DEA (deterministic, non-standard input/output treatment, super-efficiency interpretation and the limitation of the number of inputs/outputs depending on the number of observations), original models were improved by Imprecise-DEA [32], order-alpha and order-m models [33], and Monte-Carlo simulation engines for introducing probabilistic risks into DEA models [22,34,35].

Monte-Carlo simulation is a statistical technique specifically designed to introduce the risk (as an approximation of the real system uncertainty) in almost any analysis [36]. This methodology uses pseudorandom numbers that have been algorithmically produced by computers to reproduce the predefined probability distributions that represent the behaviour of system variables [37].

Although it is stated that Monte-Carlo simulation can resolve two relevant DEA drawbacks–it is a discrete model (RTE is probabilistic) and the limitation on the number of inputs/outputs depending on the number of observations- [38,39] it has not been frequently applied.

This project is part of a long-lasting collaboration between the Bizkaia department of health (Osakidetza) and an international research consortium made by the PSICOST research association (Spain), the Universidad Loyola Andalucía, and the Australian National University to improve the knowledge base for regional evidence-informed planning in MH care. This collaboration has produced the Atlases of MH Care of Bizkaia [40], and Gipuzkoa [41], and different modelling strategies for DSS to assess different patterns of care in small areas using neuronal network analysis [42] and to produce league tables of the RTE of SHAs in the Basque Country based on Monte-Carlo DEA [22].

This study presents a novel hybrid DSS (EDeS-MH) in order to support consequential priority setting of shifted/relocated resources in a regional health care system, as a demonstration of its applicability for the assessment of management interventions and its usability to generate evidence-informed recommendations at the different levels of the health care ecosystem (micro, meso and macro).

## Methods

### Setting

Bizkaia (1,156,447 inhabitants) is one of the three provinces of the Basque Country (Spain). The health department in each province has full governance capacity and centralizes health care planning and provision [43]. The MH ecosystem in Bizkaia is organised in 19 SHAs with a reference community MH centre. These SHAs are arranged in four districts with a reference general hospital for acute admissions.

### Vocabulary for the DSS

To overcome the impact of terminological variability in health system research and in the design of DSS, we have used a standard vocabulary based on the REFINEMENT glossary of terms for MH systems analysis [44]. We have added some new terms to define management interventions. Management is defined in the glossary as “the responsibility for and control of a company or organisation”.

A “management intervention” is here defined as an act of planning, organising, staffing, directing and/or controlling components of a health care ecosystem that utilises financial, human and material resources to achieve a defined health care goal. From a priority setting perspective [45], these management interventions can be classified as procedural (rules and steps to set priorities such as engagement, transparency and empowerment) and consequential (implementation of decisions and results of the priority setting such as efficiency analysis, reduction of resource waste and increase satisfaction). We have analysed consequential management interventions for shifting/reallocating resources at the micro (changes in an individual service), meso (SHAs) and macro levels (the Bizkaia MH care ecosystem). These management interventions can involve changes in the availability of services, placement capacity or workforce capacity. The obtained results can be considered relevant as a piece of knowledge for a better understanding of the evolution of the MH ecosystem.

### Procedure

The sociodemographic characteristics, the local service provision and the resource utilisation pattern have first been described for every SHA. To design useful and operable DSS, a series of steps should be followed within a systems thinking approach [46]: (a) describe the framework/model/values of the system being assessed; (b) describe the environmental characteristics of the health care ecosystem; (c) describe the agents operating in this ecosystem (consumers, professionals, teams and organisations); (d) describe the main interactions of the agents within the system; (e) apply advanced methods for knowledge discovery from data; and (f) use hybrid models that integrate operational and statistical techniques (for data analysis) as well as artificial intelligence-based techniques in order to incorporate previously formalized expert knowledge (production rules).

We combined qualitative and quantitative approaches to generate the critical knowledge to develop the EDeS-MH following these recommendations. First, we developed a Basic Mental Health Community Care Model (B-MHCCM) that can be applied to benchmarking and efficiency analysis in Spain [47].

Second, we conducted a detailed analysis of the MH care ecosystem of Bizkaia that was incorporated into the *Atlas de Salud Mental de Bizkaia* [40]. This included a description of the areas and their social and demographic characteristics. We also provided a general description of the main agents operating in this system: 1) consumers: population with mental disorders in contact with the MH services; 2) professionals; 3) care teams; and 4) organisations. The DESDE-LTC system (Description and Evaluation of Services and DirectoriEs for Long Term Care) [48] was used for the standard description of the MH service provision (professionals, care teams and organisations). DESDE-LTC uses an international classification for coding care teams in services [49] to allow comparisons across different jurisdictions. Care Teams or “Basic Stable Inputs of Care” (BSICs) are coded according to the following main types of care: “R” Residential services, “D” Day care services and “O” Outpatient services. The description included the service availability, its placement capacity (number of beds or places in day care) and the workforce capacity measured in full-time equivalents. This information was aggregated at meso (19 SHAs) and macro (Bizkaia province) levels.

Third, the interactions of different agents in the system were analysed by the resource utilization of hospital and outpatient services obtained from databases of the department of health in this jurisdiction [40].

Fourth, we incorporated experts in the knowledge discovery from data. This expert group included, for Bizkaia, the senior managers (2 people, psychiatrists), MH services managers (depending on the meeting, from 3 to 5 people; psychologists and psychiatrists) and information system managers (depending on the meeting, from 1 person to 3 people). For the design of the scenarios and for the interpretation of the variable values, experts from the Catalonia Autonomous Community and Gipuzkoa (Basque Country) were also included in the discussion groups (only MH managers; psychologists and psychiatrists). In a previous work [50] we proposed an approach called “Expert-based Collaborative Analysis” (EbCA) to generate a minimum metadata set, the EDeS-MH core, that included 57 variables for describing the structure of the MH system in 19 DMUs (SHAs). The experts followed an information-guided approach using visualisations of these variables to generate estimates of the range of appropriateness for every indicator. Using this information, 43 variables are identified as inputs (Group C-Quality of care in the REFINEMENT glossary): availability, placement capacity and workforce capacity. The remaining 14 variables were labelled as outputs (Group B-Service Utilization): hospital discharges, readmissions, average length of stay, utilization of acute and non-acute day care and, finally, utilization of non-acute outpatient care (prevalence, incidence and frequency of visits). The minimum dataset for this study is available in the Dryad Digital Repository: https://doi.org/10.5061/dryad.1qd621b.

### Design of the scenarios

The concept of scenario refers to different meaningful combinations of variables (inputs and outputs). By using the EbCA model (for checking the model structure and details see [50]), experts in MH care planning and management and service researchers designed 15 scenarios for assessing the RTE of SHAs. The first 9 described specific main types of care for each DMU: Scenario 1 (S1) acute hospital care; Scenario 2 (S2) non-acute hospital care; Scenario 3 (S3) residential community care; Scenario 4 (S4) day care health related; Scenario 5 (S5) day care; Scenario 6 (S6) outpatient care; Scenario 7 (S7) placement capacity; Scenario 8 (S8) placement capacity health related; and Scenario 9 (S9) health related workforce capacity. The remaining scenarios were designed as combinations of different main types of care according to the B-MHCCM [47]: Scenario 10 (S10) included the workforce capacity for residential, day and outpatient care; Scenario 11 (S11) included a mix of residential and health related day care with outpatient care; Scenario 12 (S12) combined acute hospital, community residential, day and outpatient types of care; Scenario 13 (S13) combined placement capacity of acute hospital, day health-non related health and outpatient care; Scenario 14 (S14) was a mix of day and residential placement and workforce capacity of residential-outpatient care; finally, Scenario 15 (S15) included a combination of health/community residential placement capacity and workforce capacity of residential-outpatient care (see Table 1 for an in-depth description of selected scenarios). For each DMU and for the MH system, the RTE was probabilistically assessed from these 15 different points of view (scenarios) in order to provide a holistic perspective of the system performance.

**Table 1:** Descriptions of the scenarios directly affected by the proposed interventions.

Scenario	Main topic of the scenario	Variables–Inputs ^a^	Variables–Outputs ^b^
**S4**	Day care health related	Acute health day care, e.g. day hospital (*TD1*^a^) Non-acute health day care, e.g. day health centre (*TD41*^a^) Acute and non-acute health day care (*ProfTotD1+D41*^a^, *ProfPsychiD1+D41*^a^, *ProfPsychoDUED1+D41*^a^, *PD1+D41*^a^*)*	Acute health day care, e.g. day hospital (*UD1*^b^) Non-acute health day care, e.g. day health centre (*UD41*^b^)
**S6**	Outpatient care	Non-acute non-mobile outpatient care, e.g. outpatient care centre (*TO8+O10*, *ProfPsychiO8+O10*, *ProfPsychoO8+O10*, *ProfDUEO8+O10*, *ProfTotO8+O10)*	Non-acute non-mobile outpatient care, e.g. outpatient care centre (*UPrevO8+O10*, *UIncO8+O10*, *UFrecO8+O10)*
**S7**	Placement capacity	Acute hospital care, e.g. acute ward (PR2) High intensity residential care, e.g. hostel (PR8+R11) Acute and non-acute health day care (PD1+D41) Day care (others), e.g. social club (PD4other) Day care (others) and work-related day care (PD4other+D2-D3)	Acute hospital care, e.g. acute ward (*UDischargesR2*, *UStayR2)* Non-acute non-mobile outpatient care, e.g. outpatient care centre (*UPrevO8+O10*, *UFrecO8+O10)*
**S8**	Placement capacity health related	Acute hospital care, e.g. acute ward (PR2) Non-acute care, e.g. sub-acute ward, non-acute crisis home (PR4-R7) Acute health day care, e.g. day hospital (*PD1*) Non-acute health day care, e.g. day health centre (*PD41*)	Acute hospital care, e.g. acute ward (*UDischargesR2*, *UStayR2*, *UReAdmissionR2)* Non-acute non-mobile outpatient care, e.g. outpatient care centre (*UPrevO8+O10*, *UFrecO8+O10)*
**S9**	Workforce capacity health related	Acute hospital care, e.g. acute ward (*ProfPsychiR2*, *ProfPsycho-DUER2*) Non-acute care, e.g. sub-acute ward, non-acute crisis home (*ProfPsychiR4-R7*, *ProfPsychoDUER4-R7)* Acute and non-acute health day care (*ProfTotD1+D41)*	Acute hospital care, e.g. acute ward (*UDischargesR2)* Non-acute non-mobile outpatient care, e.g. outpatient care centre (*UPrevO8+O10)*
**S10**	Workforce capacity total	Acute hospital care, e.g. acute ward (*ProfTotR2*) Non-acute care, e.g. sub-acute ward, non-acute crisis home (*ProfTotR4-R7)* Residential care (*ProfTotR8-R13)* Acute and non-acute health day care (*ProfTotD1+D41)* Day care (others) and work-related day care (*ProfTotD4+D2-D3)* Non-acute non-mobile outpatient care, e.g. outpatient care centre (*ProfTotO8+O10)*	Acute hospital care, e.g. acute ward (*UDischargesR2)* Non-acute non-mobile outpatient care, e.g. outpatient care centre (*UPrevO8+O10)*
**S11**	Combination of residential and day health related placement capacity and availability of outpatient care	Acute hospital care, e.g. acute ward (PR2) Non-acute care, e.g. sub-acute ward, non-acute crisis home (PR4-R7) Acute and non-acute health day care (*PD1+D41)* Non-acute non-mobile outpatient care, e.g. outpatient care centre *(TO8+O10)*	Acute hospital care, e.g. acute ward (*UDischargesR2*, *UStayR2*, *UReAdmissionR2)* Non-acute non-mobile outpatient care, e.g. outpatient care centre (*UPrevO8+O10*, *UFrecO8+O10)*
**S12**	Combination of hospital-community residential and day health related placement capacity and outpatient availability	Acute hospital care, e.g. acute ward (PR2) Residential care (*PR8-R13)* Acute and non-acute health day care (*PD1+D41)* Non-acute non-mobile outpatient care, e.g. outpatient care centre *(TO8+O10)*	Acute hospital care, e.g. acute ward (*UDischargesR2*, *UStayR2)* Non-acute non-mobile outpatient care, e.g. outpatient care centre (*UPrevO8+O10*, *UFrecO8+O10)*
**S13**	Placement capacity of acute residential, day and outpatient care	Acute hospital care, e.g. acute ward (PR2) High intensity residential care, e.g. hostel (PR8+R11) Residential care (others), e.g. supported accommodation/group homes (PR12) Acute and non-acute health day care (*PD1+D41)* Day care (others) and work-related day care (PD4other+D2-D3)	Acute hospital care, e.g. acute ward (*UDischargesR2*, *UReAdmissionR2)* Non-acute non-mobile outpatient care, e.g. outpatient care centre (*UPrevO8+O10*, *UFrecO8+O10)*
**S14**	Combination of day and residential placement and workforce capacity of residential-outpatient care	Acute hospital care, e.g. acute ward (PR2) Non-acute care, e.g. sub-acute ward, non-acute crisis home (PR4-R7) Acute and non-acute health day care (*PD1+D41)* Acute hospital care, e.g. acute ward (*ProfPsychiR2)* Non-acute care, e.g. sub-acute ward, non-acute crisis home (*ProfTotR4-R7)* Non-acute non-mobile outpatient care, e.g. outpatient care centre (*ProfTotO8+O10)*	Acute hospital care, e.g. acute ward (*UDischargesR2)* Non-acute non-mobile outpatient care, e.g. outpatient care centre (*UPrevO8+O10)*
**S15**	Combination of health-community residential placement capacity and workforce capacity of residential-outpatient care	24-h medical support hospital and residential care *(PR2-R7*, *ProfTotR2-R7)* Residential care (*PR8-R13*, *ProfTotR8-R13)* Non-acute non-mobile outpatient care, e.g. outpatient care centre (*ProfTotO8+O10)*	Acute hospital care, e.g. acute ward (*UDischargesR2*, *UReAdmissionR2)* Non-acute non-mobile outpatient care, e.g. outpatient care centre (*UPrevO8+O10)*

^a^ Availability (T): Number of MTC (R2, R4 to R7, R8 to R13, D1+D41 and O8 to O10) in the DMU. Placement capacity (P): Places or beds at the DMU. Workforce capacity (ProfPsychi, ProfPsycho, ProfDUE, ProfTot): Number of psychiatrists, psychologists, nurses and total respectively in the DMU.

^b^ Utilization (UDischarges, UReadmission, UStay, UPrev, UFrec): discharges, readmissions and length of stay for residential acute hospital care (R2); Prevalence and Incidence for Outpatient care.

### Design of the management interventions

To assess the usability of the EDeS-MH, three management interventions that involved shifting staff across different services in different SHAs of the Bizkaia MH system have been tested. These mesomanagement interventions were designed by senior managers of the Bizkaia MH system to provide a better community MH care.

Mesomanagement intervention 1: Reassigning a psychologist from an outpatient care service (coded by letter O, DESDE-LTC) located in SHA-1 (Uribe) to a day hospital (code D41) located in SHA-2 (Durango). This mesomanagement intervention modifies the structure of a day hospital that provides MH care not only to the Durango SHA but also to the Basauri, Bermeo, Galdakao and Gernika SHAs.

Mesomanagement intervention 2: Reassigning a psychiatrist from an outpatient care service (code O) located in SHA-1 (Uribe) to an outpatient service located in SHA-3 (Sestao).

Mesomanagement intervention 3: Reassigning a psychiatrist from an outpatient care service (code O8-O10) located in SHA-4 (Ercilla) to an outpatient service located in SHA-5 (Barakaldo).

The mesomanagement interventions 1, 2 and 3 directly modified many variable values in specific SHAs (Table 2). Due to the structural modification of the day hospital located in Durango, the mesomanagement intervention 1 modified some secondary variable values (causal effect) in other SHAs. In addition to these variables, the budget in all of the affected areas had to be correspondingly modified.

**Table 2:** Variables affected in the interventions.

Variables	Intervention 1	Intervention 2	Intervention 3
Inputs (resources)	Number of professionals working in outpatient care (total) [*ProfTotO8+O10*] in Uribe. Decreasing. N° of psychologists working in outpatient care [*ProfPsychoO8+O10*] in Uribe. Decreasing. N° of psychiatrists working in outpatient care [*ProfPsychiO8+O10*] in Uribe. Decreasing. N° of psychologists working in day hospital care [*ProfPsychoDUED1+D41*] in Durango. This variable integrates D1 and D41 services. Increasing. N° of professionals working in day hospital care [*ProfTotD1+D41*] in Durango. This variable integrates D1 and D41 services. Increasing. N° of professionals working in outpatient care (total) [*ProfTotO8+O10*] in Sestao. Increasing. N° of psychiatrists working in outpatient care [*ProfPsychiO8+O10*] in Sestao. Increasing. Due to the structural modification of the day hospital located in Durango, the management intervention 1 modified the following variable values: N° of psychologists working in day hospital care [ProfPsychoDUED1+D41] in Basauri. This variable integrates D1 and D41 services. Increasing. N° of professionals working in day hospital care [ProfTotD1+D41] in Basauri. This variable integrates D1 and D41 services. Increasing. N° of psychologists working in day hospital care [ProfPsychoDUED1+D41] in Bermeo. This variable integrates D1 and D41 services. Increasing. N° of professionals working in day hospital care [ProfTotD1+D41] in Bermeo. This variable integrates D1 and D41 services. Increasing. N° of psychologists working in day hospital care [ProfPsychoDUED1+D41] in Galdakao. This variable integrates D1 and D41 services. Increasing. N° of professionals working in day hospital care [ProfTotD1+D41] in Galdakao. This variable integrates D1 and D41 services. Increasing. N° of psychologists working in day hospital care [ProfPsychoDUED1+D41] in Gernika. This variable integrates D1 and D41 services. Increasing. N° of professionals working in day hospital care [ProfTotD1+D41] in Gernika. This variable integrates D1 and D41 services. Increasing.	Number of professionals working in outpatient care (total) [*ProfTotO8+O10*] in Uribe. Decreasing. N° of psychologists working in outpatient care [*ProfPsychoO8+O10*] in Uribe. Decreasing. N° of psychiatrists working in outpatient care [*ProfPsychiO8+O10*] in Uribe. Decreasing. N° of psychologists working in day hospital care [*ProfPsychoDUED1+D41*] in Durango. This variable integrates D1 and D41 services. Increasing. N° of professionals working in day hospital care [*ProfTotD1+D41*] in Durango. This variable integrates D1 and D41 services. Increasing. N° of professionals working in outpatient care (total) [*ProfTotO8+O10*] in Sestao. Increasing. N° of psychiatrists working in outpatient care [*ProfPsychiO8+O10*] in Sestao. Increasing.	N° of professionals working in outpatient care (total) [ProfTotO8+O10] in Ercilla. Decreasing. N° of psychiatrists working in outpatient care [ProfPsychiO8+O10] in Ercilla. Decreasing. N° of professionals working in outpatient care (total) [ProfTotO8+O10] in Barakaldo. Increasing. N° of psychiatrists working in outpatient care [ProfPsychiO8+O10] in Barakaldo. Increasing.
Outputs (outcomes)	Use of day hospital care [UD41]. Increasing, constant or decreasing depending on the affected SHA. Frequentation in outpatient care [UFrecO8+O10]. Increasing or constant depending on the affected SHA. N° of places in day hospital care [PD41]. Increasing or decreasing depending on the affected SHA. N° of places in day care [PD1+D41]. Increasing or decreasing depending on the SHA.	Frequentation in outpatient care [UFrecO8+O10]. Increasing or constant depending on the affected SHA.	Frequentation in outpatient care [UFrecO8+O10]. Increasing or constant depending on the affected SHA.

These modifications generated real expectations (in terms of expected outcomes) for decision makers (Table 2).

### Randomization of the original data (understanding the environmental and structural uncertainties)

To integrate into the model the uncertainties of the MH systems, the majority of the original dataset was randomized by using standard triangular statistical distributions (1).

$$T[w_{tl}x_{i},w_{tr}x_{i}]$$ (1)

being, *x*_*i*_ the original value of the variable and *w*_*tl*_ and *w*_*tr*_ the weights that define the range of the triangular distribution (*w*_*tl*_ = 0.9 and *w*_*tr*_ = 1.1). For example, if the variable value of *TD1* is 0.4398 in Bermeo SHA, the corresponding triangular distribution is: minimum value 0.9x0.4398 = 0.3958, modal value 0.4398 and maximum value 1.1x0.4398 = 0.4838, T[0.3958, 0.4398, 0.4838]. This procedure is deeply explained in a previous work [22].

For the variable *UFrecO8+O10* (output in many scenarios) in Barakaldo, Ercilla, Sestao and Uribe, uniform (rectangular) statistical distributions (2) were used:

$${U[x}_{i},w_{ur}x_{i}]$$ (2)

being, *w*_*ur*_ the weight that define the range of the uniform distribution (*w*_*ur*_ = 1.1). For example, if the variable value of *UFrecO8+O10* in Bermeo is 439.12, the corresponding rectangular (uniform) is: minimum value 439.12 and maximum value 1.1x439.12 = 483.03, U[439.12, 483.03]. This procedure is deeply explained in a previous work [22].

In the end, the original 19×57 dataset (19 SHAs and 57 variables) was transformed into a 19×57 statistical distributions (triangular or uniform) matrix. This structure fed, in an iterative process, the Monte-Carlo simulation engine for the design of the final dataset before carrying out the variable values interpretation (fuzzy inference engine) and RTE assessment. Once RTE scores were calculated for a specific dataset, results were stored in a solution pool and the process started again by selecting at random a new dataset using the Monte-Carlo simulation engine.

The proposed mesomanagement interventions directly involved 47 variable values (statistical distributions): 4.34%, minor changes in the dataset structure. Nevertheless, the numbers of affected SHAs 8/19 (42.11%) and scenarios 11/15 (73.33%, S4, S6-S9, S11-S15) were very relevant. Regarding the workforce capacity, only three persons (2 psychiatrists and 1 psychologist) were reassigned (0.32% of the total personnel of Bizkaia’s MH system).

### The fuzzy inference engine prototype

Once the Monte-Carlo simulation engine selects at random a variable value *x*_*i*_ according to the statistical distribution selected for *X*_*i*_ (triangular or uniform), it is interpreted according to the B-MHCCM. So, *x*_*i*_ is transformed into $x_{i}^{int}$ as a result of a function: $x_{i}^{int}=f(x_{i}|x_{i}^{left},x_{i}^{right})$ that represents a recursive relationship (the interpreted value depends on the original value, once $x_{i}^{left}$ and $x_{i}^{right}$ are defined: the range for interpreting the appropriateness degree of *x*_*i*_). This function *f* is different at the left hand side of $x_{i}^{left}$, within the range and at the right hand side of $x_{i}^{right}$, but it is always linear and monotone and it increases/decreases the original value *x*_*i*_ according the explicit expert knowledge formalized in the B-MHCCM. For example, a variable value for *TR12* (input, availability of community residential care–rate per 100.000 inhabitants) can be considered “appropriate” within the range (0.9753, 1.0979) and inside it the more centred the value the more appropriate. In this specific case, the original value is transformed by the linear monotone function: $x_{i}^{int}=(x_{i}^{right}-x_{i}^{left})-x_{i}$, being $x_{i}^{right}=1.0979$ and $x_{i}^{left}=0.9753$.

Under real conditions, $x_{i}^{left}$ and $x_{i}^{right}$ depend on the values of other variables in the dataset according to a causal model that represents the B-MHCCM. This circumstance means that the interpretation of the variable *X*_*i*_ depends on the value of the rest of the variables. The fuzzy inference engine prototype takes into consideration that Residential care (R) provision depends on Day care (D) and Outpatient care (O) provision. This relationship is defined through linking $x_{i}^{left}$ and $x_{i}^{right}$ for each variable in R to the values of D and O calculated by the fuzzy inference engine in a range [0, 100] (0: no availability of the corresponding services and 100: the availability is maximum). These D and O values are determined by using the values of the respective professional, places/beds and availability variables (rates per 100.000 inhabitants).

Each variable in the fuzzy inference engine prototype is limited to 5 normal fuzzy sets with sum equal to 1 in a range defined by a minimum (usually 0) and a maximum (defined by the experts) specific for each variable. The semantic labels associated to each fuzzy set are: very little (Z function), little (triangular), standard (triangular), much (triangular) and very much (Z function). The fuzzification process is standard once the original value *x*_*i*_ is known. Due to the structure of the fuzzy sets, each *x*_*i*_ can only have 1 or 2 membership degrees greater than cero and their sum is always equal to 1. In all the cases, the standard product-sum gravity method is used for the defuzzyfication process [51].

The structure of the production (fuzzy) rules is standard. For example, IF O is “Very little” and D is “Little” THEN $x_{TR12}^{left}$ should be “Much”. According to this rule, $x_{TR12}^{left}$ and $x_{TR12}^{right}$ will be displaced to the right. The fuzzy inference engine firstly calculates $x_{i}^{left}$ in a range defined by the experts. In this range, the prototype takes into consideration 5 fuzzy sets (with the same semantic labels: very little, little, standard, much and very much). Once the new $x_{i}^{{left}^{\prime }}$ value is calculated (defuzzyfication process) the new right limit for the variable value interpretation is calculated as follows: $x_{i}^{{right}^{\prime }}=x_{i}^{{left}^{\prime }}+(x_{i}^{right}-x_{i}^{left})$. In conclusion, the interpretation of each variable in R depends on D and O provision.

### Relative Technical Efficiency (RTE)

RTE can be assessed from both a constant or a Variable Returns to Scale (VRS). The former approach implies that any input/output variation has a proportional output/input change [21]. In the latter, which is more realistic, this does not occur [52].

RTE can also be calculated from an input or output point of view (input-oriented or output-oriented techniques) [53]. In the first approach, the model tries to minimize the input consumption when maintaining the output production constant and is used when the situation is focused on input management. In the second, it tries to maximize the output production when maintaining the input consumption constant and is used for output management purposes.

The hybrid EDeS-MH DSS combines the following methodologies:

- Monte-Carlo simulation (in order to carry out this technique a Monte-Carlo simulation engine was designed and developed) for analysing the environmental and structural uncertainties of the MH system.
- Artificial intelligence by using “IF…Then” rule-based criteria to introduce the expert´s opinion, based on the B-MHCCM.
- DEA, both input and output-oriented and variable returns to scale for RTE assessment.
- Basic Statistics: Kolmogorov-Smirnov test, T-test and Levene´s Test for the assessment of the intervention impact.
- Stability and Entropy for analysing the pre and post-intervention stability and uncertainty of the MH system.

### DSS structure (pseudocode)

In order to assess the RTE of each scenario, both the fuzzy inference engine (to interpret the variable values according to the B-MHCCM) and the operational model (to calculate RTE scores) are embedded in the Monte-Carlo simulation engine (to integrate the inner uncertainty of the environment). The process is iterative and RTE scores are saved in a solution pool. The proposed pseudocode is:

1: **DSS procedure**

2:  The solution pool (SP) is cleaned

3:  **For** *j* = 1 to *n*_*sim*_ **do** → being *j* the simulation number and *n*_*sim*_ the maximum number of simulations

4:   **For** *i* = 1 to *n*_*sce*_ **do** → being *i* the scenario number and *n*_*sce*_ the maximum number of scenarios

5:    **For** *k* = 1 to *n*_*dmu*_ **do** → being *k* the SHA (DMU) number and *n*_*dmu*_ the number of SHA

6:     Original dataset **S**_**ijk**_ → the Monte-Carlo simulation engine selects at random a value for each simulation, variable and SHA *k* according to their specific statistical distributions.

7:     Calculate **D**_**ijk**_ and **O**_**ijk**_ → the fuzzy inference engine determines the day care and outpatient care fuzzy sets and membership degrees for each SHA

8:     Calculate $x_{ijk}^{left}$ and $x_{ijk}^{right}$ → the fuzzy inference engine determines the range for variable value interpretation according to the B-MHCCM

9:     Interpreted dataset **I**_**ijk**_ → the original dataset **S**_**ijk**_ are interpreted by the fuzzy inference engine according to the B-MHCCM

10:      Calculate **RTE**_**ijk**_ scores → variable returns to scale Data Envelopment Analysis (DEA) model (both input and output-oriented)

11:      Save **RTE**_**ijk**_ scores in the solution pool

12:    **end for**

13:   **end for**

14: **end for**

15: **end procedure**

This pseudocode is published in: dx.doi.org/10.17504/protocols.io.vyke7uw.

**Fig 1:**
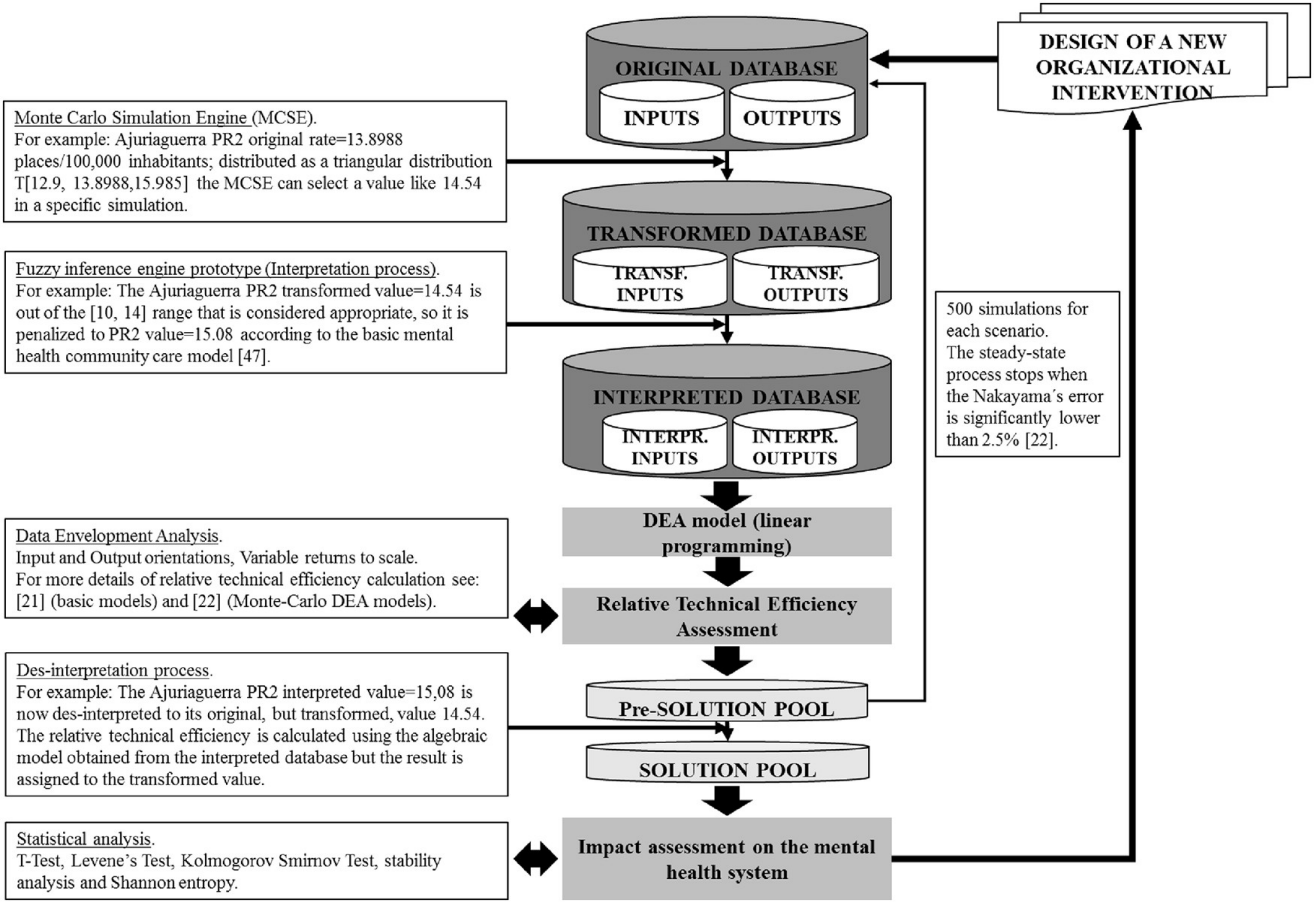
Analysis of an organizational intervention: A flowchart.

For each SHA and scenario (15), 500 simulations were carried out. Therefore, each scenario was analysed by 9,500 simulations (19×500) and the global RTE of the Bizkaia MH system was assessed by 142,500 simulations (19×15×500). This process was repeated 4 times using the DEA-VRS assessing (i) pre-management interventions and input-oriented RTE, (ii) pre-management interventions and output-oriented RTE, (iii) post-management interventions and input-oriented RTE and (iv) post-management interventions and output-oriented RTE (the whole process is described in Fig 1).

**Fig 2:**
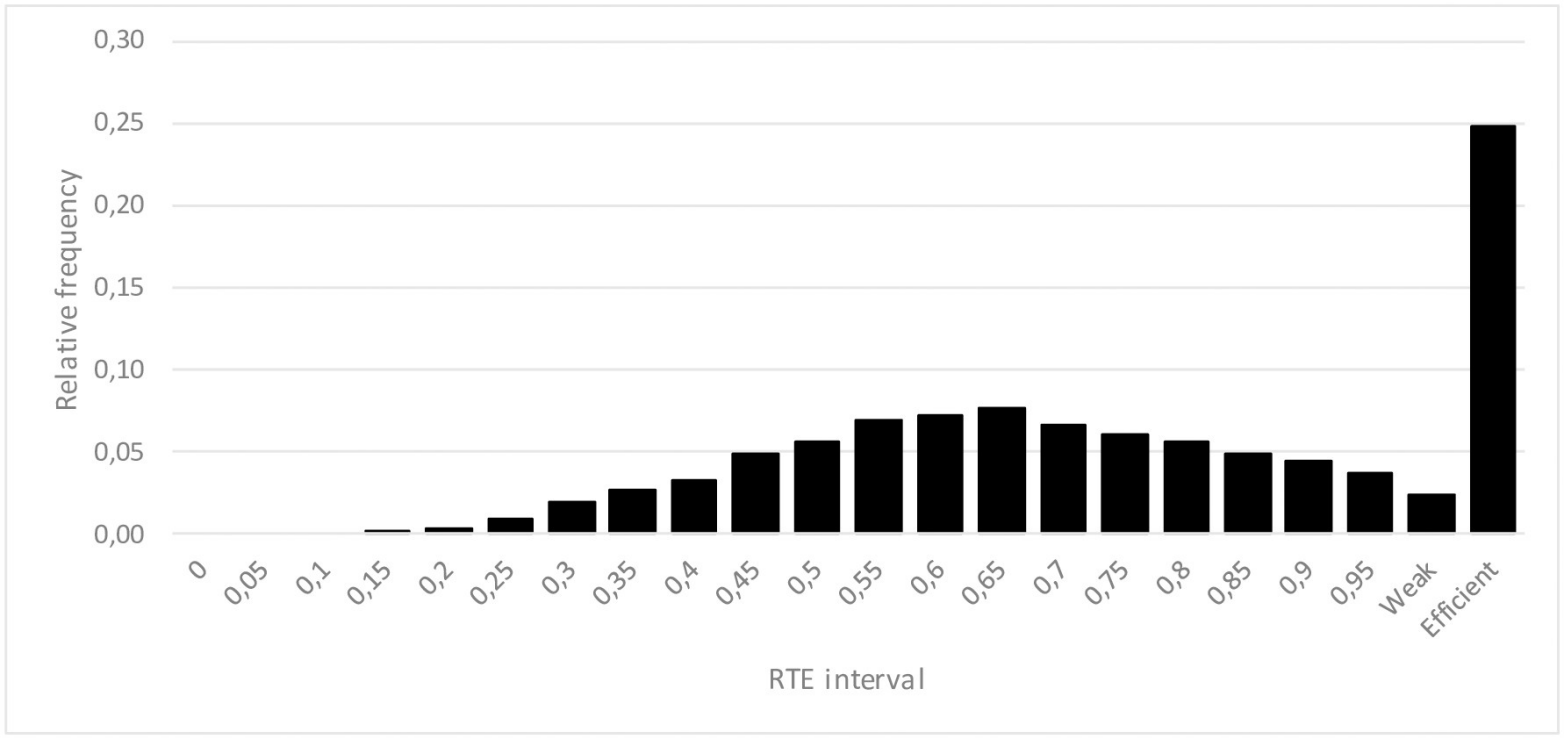
An example of resulting RTE statistical distribution for a specific DMU. The first interval (at the left hand side) is [0, 0.05), the second is [0.05, 0.1), …, the antepenultimate is [0.95, 1), the penultimate represents the simulations where RTE = 1 but the sum of slacks are different to zero (weakly efficient) and, finally, the last interval represents the simulations where RTE = 1 and the sum of the slacks is equal to zero (completely efficient).

In all the assessed cases, the calculated RTE is a probabilistic distribution (Fig 2) that can be analysed by both a frequency analysis and statistical estimators:

- Probability of being efficient (*P*_*RTE*⩵1_).
- Probability of being weakly efficient (*P*_*RTE*≅1_)
- Probability of being inefficient (*P*_*RTE*<1_).
- Efficiency average $\left(\overset{-}{RTE}\right)$.
- Efficiency standard deviation $\left(\overset{-}{\sigma_{RTE}}\right)$.
- Efficiency error $\left(\overset{-}{\varepsilon_{RTE}}\right)$.
- Efficiency error $\left(\overset{-}{{\%}_{RTE}}\right)$.
- Probability of having an RTE score greater than *rte*_*1*_ and lower than *rte*_*2*_.
- Probability of having an RTE score greater than *rte*_*3*_.

After an intervention or policy, the initial RTE statistical distribution can vary, significantly or not, depending on the resulting impact.

### Stability and entropy analysis

The resulting RTE statistical distributions were additionally analysed using the stability and entropy indicators. Stability aims to assess (in a [0, 100] range; 0: completely unstable and 100: completely stable) if the RTE statistical distribution is more or less sensitive to change when the input/output values change. A system can be considered stable when data changes (that represent structural changes) do not significantly modify RTE scores (the efficiency of the system is not sensitive to structural changes). In contrast, the system is unstable when small data changes vary the RTE scores dramatically (the efficiency of the system is highly dependent on small structural changes). DMU final stability (*stab*) has two components.

1. Stability due to the number of RTE intervals with probability (interval stability of DMU *i*, *intstab*_*i*_). A DMU is completely stable (*intstab*_*i*_ = 100) if all the probability is concentrated in only one RTE interval. A DMU is completely unstable (*intstab*_*i*_ = 0) when the probability is distributed in all the RTE intervals or it is concentrated in the first and the last interval.
2. Stability due to the concentration of the density of probability (density stability of DMU *i*, *denstab*_*i*_). A DMU is completely stable (*denstab*_*i*_ = 100) when all (or the majority) of the probability is concentrated in only one RTE interval, independent of where the rest is distributed. A DMU is completely unstable (*denstab*_*i*_ = 0) when all (or the majority) of the probability is concentrated in all or a much separated RTE intervals.

The interval stability of the DMU *i* (*intstab*_*i*_) is calculated in the following way (3):

$${intstab}_{i}=100-100\times ({nint}_{i}-1)/(inttot-1)$$ (3)

where *nint*_*i*_ is the number of RTE intervals in the frequency analysis that have probability greater than zero and *inttot* is the total number of intervals defined in the frequency analysis.

The density stability of the DMU *i* (*denstab*_*i*_) is calculated in the following way (4):

$${denstab}_{i}=100\times [\frac{\text{ln}(\frac{{acprob}_{i}\times 100}{{nintprob}_{i}})-minln}{\left(maxln-minln\right)}]$$ (4)

where *acprob*_*i*_ is the first accumulated probability strictly greater than a predefined probability (*prob*), *nintprob*_*i*_ is the number of intervals needed to reach *acprob*_*i*_, *minln* is the minimum feasible value for the logarithm, and *maxln* is the maximum feasible value for the logarithm.

**Fig 3:**
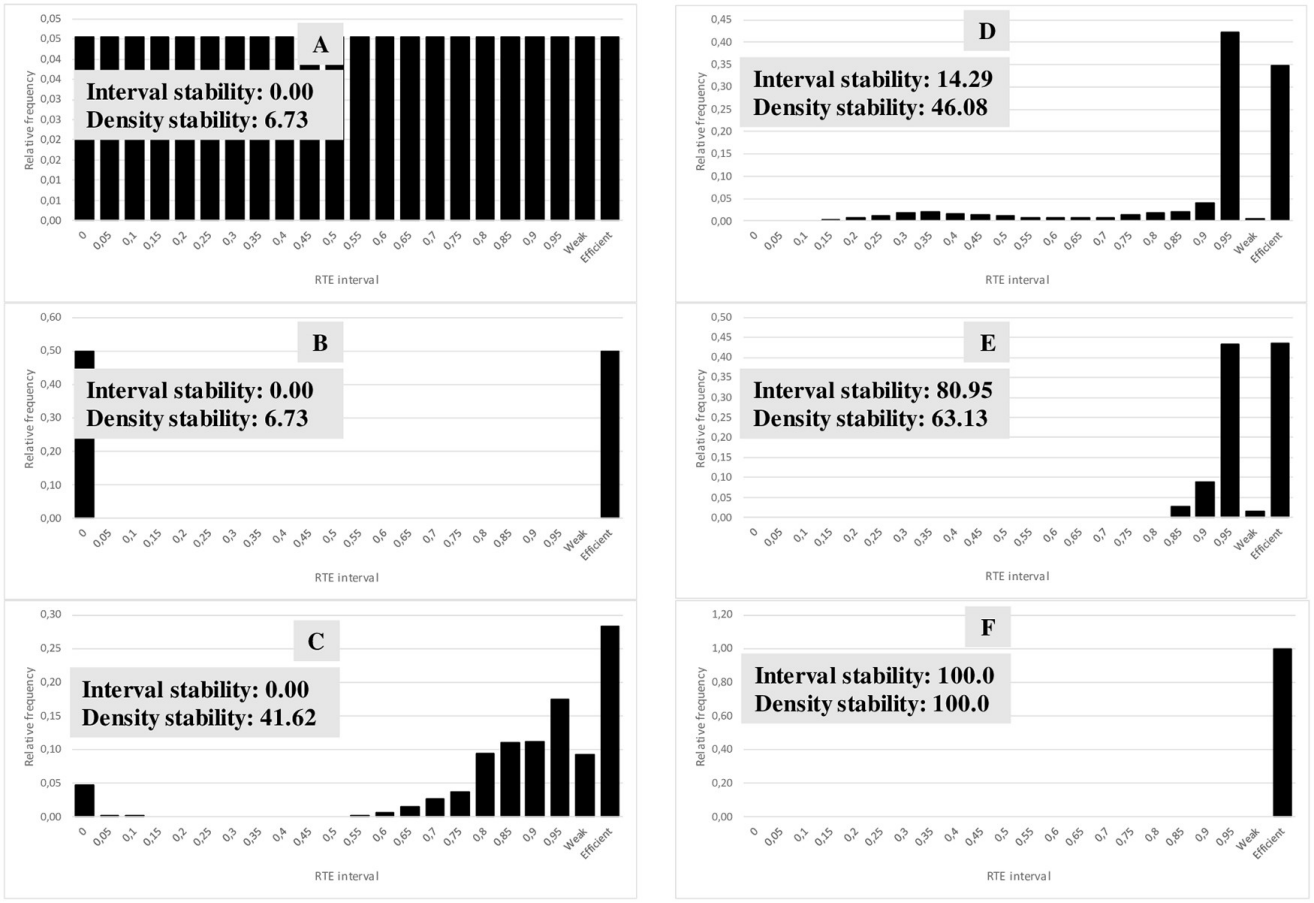
Some examples of different RTE statistical distributions and their corresponding stabilities (*inttot* = 22, prob = 0.8, *minln* = 1.2909 and *maxln* = 4.6051). For the example in E: *ninti* = 5, *acprobi* = 0.88, *nintprobi* = 3, *wint* = 0.5 and *wden* = 0.5. Finally, the resulting *stabE* = 72.04 (likely stable).

**Fig 4:**
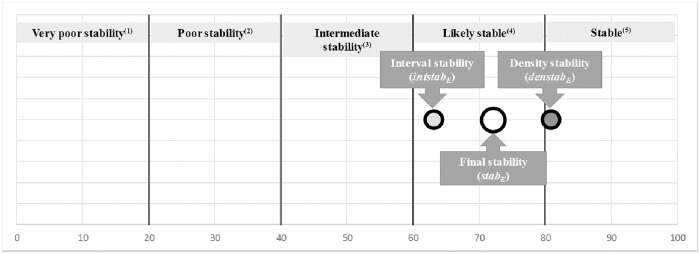
Interval stability (*intstab*_*E*_ = 80.95, circle with the darker background), density stability (*denstab*_*E*_ = 63.13, circle with the light grey) and final stability (*stab*_*E*_ = 72.04, greater circle without any background colour) for the DMU shown in the example (DMU E) in Fig 3 (*w*_*int*_ = 0.5 and *w*_*den*_ = 0.5). (1) Small changes in data values (generated by the Monte Carlo simulation engine or real ones) can change RTE scores dramatically. Decision makers should be awarded. (2) Small changes in data values (generated by the Monte Carlo simulation engine or real ones) can change RTE scores a lot. Decision makers should be awarded. (3) Changes in data values (generated by the Monte Carlo simulation engine or real ones) could change RTE scores not so much. Decision makers should be awarded. (4) Changes in data values (generated by the Monte Carlo simulation engine or real ones) do not change RTE scores a lot. (5) Changes in data values (generated by the Monte Carlo simulation engine or real ones) do not change RTE (changes, if exist, are very small).

The final stability of the DMU *i* (*stab*_*i*_) results of a weighted sum (5) of interval stability and density stability is as follows:

$${stab}_{i}=w_{int}\times {intstab}_{i}+w_{den}\times {denstab}_{i}$$ (5)

where *w*_*int*_ is the weight selected for the interval stability and *w*_*den*_ is the corresponding weight for the density stability. For a better understanding of the results (Figs 3 and 4), it could be necessary (but it is not mandatory) that: *w*_*int*_ + *w*_*den*_ = 1.

The DMU entropy (*entr*_*i*_) is calculated using (6), the Shannon’s entropy, and represents the degree of uncertainty included in the RTE statistical distribution [54]:

$${entr}_{i}=\sum_{h=1}^{nint}\left(-1\times {freq}_{h}\times {\text{log}}_{2}{freq}_{h}\right)$$ (6)

where *nin*_*t*_ is the number of intervals assessed in the frequency analysis and *freq*_*h*_ is the relative frequency in the interval *h*.

## Results

### Whole RTE assessment: Initial situation (“A”)

#### RTE results

Entire System (pre-management interventions). From an input management perspective (input-oriented DEA-VRS), the Bizkaia MH system is likely efficient. The probability of being efficient (*P*_*RTE*⩵1_) is 0.2040, the RTE average $\left(\overset{-}{RTE}\right)$ is 0.7831, and the probability of having an *RTE* greater than 0.75 is 0.6240 (Table 3, “Pre-Interventions” column).

**Table 3:** Impact of the meso-interventions (input-oriented results).

Unit of analysis	Results	Pre-Interventions	Post-Interventions	Variation (%)
**Global System**	Probability of being efficient^(1)^ (*P*_*RTE*⩵1_):	0.2040	0.2047	0.34
	Efficiency average $\left(\overset{-}{RTE}\right)$:	0.7831	0.7857	0.33
	Efficiency standard deviation^(2)^$\left(\overset{-}{\sigma_{RTE}}\right)$:	0.001161	0.001363	17.40
	Efficiency error^(2)^$\left(\overset{-}{\varepsilon_{RTE}}\right)$:	0.001444	0.001695	17.40
	Probability of having a RTE score greater than 0.75:	0.6240	0.6281	0.66
**S4**	Probability of being efficient^(1)^(*P*_*RTE*⩵1_):	0.2324	0.2280	-1.90
	Efficiency average $\left(\overset{-}{RTE}\right)$:	0.9357	0.9362	0.06
	Efficiency standard deviation^(2)^$\left(\overset{-}{\sigma_{RTE}}\right)$:	0.001708	0.001583	-7.30
	Efficiency error^(2)^$\left(\overset{-}{\varepsilon_{RTE}}\right)$:	0.002123	0.001968	-7.30
	Probability of having a RTE score greater than 0.75:	0.9260	0.9311	0.51
**S6**	Probability of being efficient^(1)^(*P*_*RTE*⩵1_):	0.2159	0.2159	0.00
	Efficiency average $\left(\overset{-}{RTE}\right)$:	0.8069	0.8059	-0.12
	Efficiency standard deviation^(2)^$\left(\overset{-}{\sigma_{RTE}}\right)$:	0.007061	0.003662	-48.14
	Efficiency error^(2)^$\left(\overset{-}{\varepsilon_{RTE}}\right)$:	0.008778	0.004553	-48.14
	Probability of having a RTE score greater than 0.75:	0.6422	0.6563	1.41
**S7**	Probability of being efficient^(1)^(*P*_*RTE*⩵1_):	0.2363	0.2488	5.30
	Efficiency average $\left(\overset{-}{RTE}\right)$:	0.7391	0.7563	2.34
	Efficiency standard deviation^(2)^$\left(\overset{-}{\sigma_{RTE}}\right)$:	0.008573	0.005478	-36.10
	Efficiency error^(2)^$\left(\overset{-}{\varepsilon_{RTE}}\right)$:	0.010658	0.006811	-36.10
	Probability of having a RTE score greater than 0.75:	0.5033	0.5199	1.66
**S8**	Probability of being efficient^(1)^(*P*_*RTE*⩵1_):	0.2859	0.2858	-0.04
	Efficiency average $\left(\overset{-}{RTE}\right)$:	0.8541	0.8473	-0.79
	Efficiency standard deviation^(2)^$\left(\overset{-}{\sigma_{RTE}}\right)$:	0.001843	0.002502	35.77
	Efficiency error^(2)^$\left(\overset{-}{\varepsilon_{RTE}}\right)$:	0.002291	0.003110	35.77
	Probability of having a RTE score greater than 0.75:	0.7555	0.7344	-2.11
**S9**	Probability of being efficient^(1)^(*P*_*RTE*⩵1_):	0.1447	0.1422	-1.75
	Efficiency average $\left(\overset{-}{RTE}\right)$:	0.8467	0.8440	-0.31
	Efficiency standard deviation^(2)^$\left(\overset{-}{\sigma_{RTE}}\right)$:	0.002497	0.004550	82.22
	Efficiency error^(2)^$\left(\overset{-}{\varepsilon_{RTE}}\right)$:	0.003104	0.005657	82.22
	Probability of having a RTE score greater than 0.75:	0.7074	0.7019	-0.55
**S10**	Probability of being efficient^(1)^(*P*_*RTE*⩵1_):	0.2017	0.1882	-6.68
	Efficiency average $\left(\overset{-}{RTE}\right)$:	0.7836	0.7836	0.00
	Efficiency standard deviation^(2)^$\left(\overset{-}{\sigma_{RTE}}\right)$:	0.005560	0.005940	6.83
	Efficiency error^(2)^$\left(\overset{-}{\varepsilon_{RTE}}\right)$:	0.006913	0.007384	6.83
	Probability of having a RTE score greater than 0.75:	0.5871	0.5844	-0.26
**S11**	Probability of being efficient^(1)^(*P*_*RTE*⩵1_):	0.2403	0.2477	3.07
	Efficiency average $\left(\overset{-}{RTE}\right)$:	0.7756	0.7908	1.96
	Efficiency standard deviation^(2)^$\left(\overset{-}{\sigma_{RTE}}\right)$:	0.003327	0.004019	20.80
	Efficiency error^(2)^$\left(\overset{-}{\varepsilon_{RTE}}\right)$:	0.004136	0.004996	20.80
	Probability of having a RTE score greater than 0.75:	0.6154	0.6356	2.02
**S12**	Probability of being efficient^(1)^(*P*_*RTE*⩵1_):	0.2244	0.2264	0.89
	Efficiency average $\left(\overset{-}{RTE}\right)$:	0.7681	0.7614	-0.87
	Efficiency standard deviation^(2)^$\left(\overset{-}{\sigma_{RTE}}\right)$:	0.004239	0.008103	91.15
	Efficiency error^(2)^$\left(\overset{-}{\varepsilon_{RTE}}\right)$:	0.005270	0.010074	91.15
	Probability of having a RTE score greater than 0.75:	0.5642	0.5514	-1.28
**S13**	Probability of being efficient^(1)^(*P*_*RTE*⩵1_):	0.3113	0.3244	4.23
	Efficiency average $\left(\overset{-}{RTE}\right)$:	0.8040	0.8124	1.04
	Efficiency standard deviation^(2)^$\left(\overset{-}{\sigma_{RTE}}\right)$:	0.003734	0.001781	-52.30
	Efficiency error^(2)^$\left(\overset{-}{\varepsilon_{RTE}}\right)$:	0.004643	0.002214	-52.30
	Probability of having a RTE score greater than 0.75:	0.6264	0.6435	1.71
**S14**	Probability of being efficient^(1)^(*P*_*RTE*⩵1_):	0.2045	0.2041	-0.21
	Efficiency average $\left(\overset{-}{RTE}\right)$:	0.7653	0.7766	1.49
	Efficiency standard deviation^(2)^$\left(\overset{-}{\sigma_{RTE}}\right)$:	0.003330	0.002666	-19.94
	Efficiency error^(2)^$\left(\overset{-}{\varepsilon_{RTE}}\right)$:	0.004140	0.003315	-19.94
	Probability of having a RTE score greater than 0.75:	0.5360	0.5666	3.06
**S15**	Probability of being efficient^(1)^(*P*_*RTE*⩵1_):	0.1479	0.1440	-2.63
	Efficiency average $\left(\overset{-}{RTE}\right)$:	0.6128	0.6158	0.50
	Efficiency standard deviation^(2)^$\left(\overset{-}{\sigma_{RTE}}\right)$:	0.007666	0.006536	-14.74
	Efficiency error^(2)^$\left(\overset{-}{\varepsilon_{RTE}}\right)$:	0.009530	0.008126	-14.74
	Probability of having a RTE score greater than 0.75:	0.3289	0.3314	0.24

^(1)^ 7500 simulations.

^(2)^ On average.

^(3)^ On average calculated taking into consideration the RTE average.

From an output management perspective (output-oriented DEA-VRS), the system is efficient. The *P*_*RTE*⩵1_ = 0.2188, $\overset{-}{RTE}=0.8812$, and the probability of having an *RTE* greater than 0.75 is 0.8805 (Table 4, “Pre-Interventions” column).

**Table 4:** Impact of the meso-interventions (output-oriented results).

Unit of analysis	Results	Pre-Interventions	Post-Interventions	Variation (%)
**Global System**	Probability of being efficient^(1)^(*P*_*RTE*⩵1_):	0.2188	0.2172	-0.72
	Efficiency average $\left(\overset{-}{RTE}\right)$:	0.8812	0.8806	-0.07
	Efficiency standard deviation^(2)^$\left(\overset{-}{\sigma_{RTE}}\right)$:	0.001096	0.001302	18.79
	Efficiency error^(2)^$\left(\overset{-}{\varepsilon_{RTE}}\right)$:	0.001362	0.001618	18.79
	Probability of having a RTE score greater than 0.75:	0.8805	0.8803	-0.02
**S4**	Probability of being efficient^(1)^(*P*_*RTE*⩵1_):	0.2335	0.2328	-0.27
	Efficiency average $\left(\overset{-}{RTE}\right)$:	0.9194	0.9190	-0.04
	Efficiency standard deviation^(2)^$\left(\overset{-}{\sigma_{RTE}}\right)$:	0.003094	0.002681	-13.36
	Efficiency error^(2)^$\left(\overset{-}{\varepsilon_{RTE}}\right)$:	0.003847	0.003333	-13.36
	Probability of having a RTE score greater than 0.75:	0.9226	0.9182	-0.44
**S6**	Probability of being efficient^(1)^(*P*_*RTE*⩵1_):	0.2323	0.2404	3.49
	Efficiency average $\left(\overset{-}{RTE}\right)$:	0.8408	0.8416	0.09
	Efficiency standard deviation^(2)^$\left(\overset{-}{\sigma_{RTE}}\right)$:	0.006258	0.003290	-47.42
	Efficiency error^(2)^$\left(\overset{-}{\varepsilon_{RTE}}\right)$:	0.007780	0.004091	-47.42
	Probability of having a RTE score greater than 0.75:	0.7464	0.7487	0.23
**S7**	Probability of being efficient^(1)^(*P*_*RTE*⩵1_):	0.2589	0.2579	-0.41
	Efficiency average $\left(\overset{-}{RTE}\right)$:	0.9146	0.9144	-0.01
	Efficiency standard deviation^(2)^$\left(\overset{-}{\sigma_{RTE}}\right)$:	0.002161	0.002601	20.34
	Efficiency error^(2)^$\left(\overset{-}{\varepsilon_{RTE}}\right)$:	0.002687	0.003234	20.34
	Probability of having a RTE score greater than 0.75:	0.9369	0.9367	-0.02
**S8**	Probability of being efficient^(1)^(*P*_*RTE*⩵1_):	0.3118	0.3043	-2.40
	Efficiency average $\left(\overset{-}{RTE}\right)$:	0.9259	0.9232	-0.29
	Efficiency standard deviation^(2)^$\left(\overset{-}{\sigma_{RTE}}\right)$:	0.002757	0.004586	66.33
	Efficiency error^(2)^$\left(\overset{-}{\varepsilon_{RTE}}\right)$:	0.003428	0.005701	66.33
	Probability of having a RTE score greater than 0.75:	0.9375	0.9344	-0.31
**S9**	Probability of being efficient^(1)^(*P*_*RTE*⩵1_):	0.1616	0.1534	-5.08
	Efficiency average $\left(\overset{-}{RTE}\right)$:	0.8551	0.8519	-0.37
	Efficiency standard deviation^(2)^$\left(\overset{-}{\sigma_{RTE}}\right)$:	0.001859	0.002693	44.87
	Efficiency error^(2)^$\left(\overset{-}{\varepsilon_{RTE}}\right)$:	0.002311	0.003348	44.87
	Probability of having a RTE score greater than 0.75:	0.8591	0.8547	-0.43
**S10**	Probability of being efficient^(1)^(*P*_*RTE*⩵1_):	0.2052	0.1963	-4.31
	Efficiency average $\left(\overset{-}{RTE}\right)$:	0.8616	0.8613	-0.04
	Efficiency standard deviation^(2)^$\left(\overset{-}{\sigma_{RTE}}\right)$:	0.007605	0.004822	-36.60
	Efficiency error^(2)^$\left(\overset{-}{\varepsilon_{RTE}}\right)$:	0.009455	0.005995	-36.60
	Probability of having a RTE score greater than 0.75:	0.8700	0.8754	0.54
**S11**	Probability of being efficient^(1)^(*P*_*RTE*⩵1_):	0.2649	0.2618	-1.19
	Efficiency average $\left(\overset{-}{RTE}\right)$:	0.9311	0.9290	-0.23
	Efficiency standard deviation^(2)^$\left(\overset{-}{\sigma_{RTE}}\right)$:	0.001243	0.004267	243.35
	Efficiency error^(2)^$\left(\overset{-}{\varepsilon_{RTE}}\right)$:	0.001545	0.005305	243.35
	Probability of having a RTE score greater than 0.75:	0.9447	0.9432	-0.16
**S12**	Probability of being efficient^(1)^(*P*_*RTE*⩵1_):	0.2499	0.2448	-2.02
	Efficiency average $\left(\overset{-}{RTE}\right)$:	0.9290	0.9272	-0.18
	Efficiency standard deviation^(2)^$\left(\overset{-}{\sigma_{RTE}}\right)$:	0.001828	0.002106	15.20
	Efficiency error^(2)^$\left(\overset{-}{\varepsilon_{RTE}}\right)$:	0.002273	0.002618	15.20
	Probability of having a RTE score greater than 0.75:	0.9573	0.9522	-0.51
**S13**	Probability of being efficient^(1)^(*P*_*RTE*⩵1_):	0.3445	0.3532	2.51
	Efficiency average $\left(\overset{-}{RTE}\right)$:	0.9299	0.9293	-0.07
	Efficiency standard deviation^(2)^$\left(\overset{-}{\sigma_{RTE}}\right)$:	0.005803	0.001284	-77.88
	Efficiency error^(2)^$\left(\overset{-}{\varepsilon_{RTE}}\right)$:	0.007214	0.001596	-77.88
	Probability of having a RTE score greater than 0.75:	0.9441	0.9444	0.03
**S14**	Probability of being efficient^(1)^(*P*_*RTE*⩵1_):	0.2141	0.2125	-0.74
	Efficiency average $\left(\overset{-}{RTE}\right)$:	0.8596	0.8621	0.29
	Efficiency standard deviation^(2)^$\left(\overset{-}{\sigma_{RTE}}\right)$:	0.005651	0.002801	-50.43
	Efficiency error^(2)^$\left(\overset{-}{\varepsilon_{RTE}}\right)$:	0.007025	0.003483	-50.43
	Probability of having a RTE score greater than 0.75:	0.8640	0.8706	0.66
**S15**	Probability of being efficient^(1)^(*P*_*RTE*⩵1_):	0.1724	0.1681	-2.50
	Efficiency average $\left(\overset{-}{RTE}\right)$:	0.8781	0.8773	-0.10
	Efficiency standard deviation^(2)^$\left(\overset{-}{\sigma_{RTE}}\right)$:	0.004450	0.004686	5.30
	Efficiency error^(2)^$\left(\overset{-}{\varepsilon_{RTE}}\right)$:	0.005533	0.005826	5.30
	Probability of having a RTE score greater than 0.75:	0.9064	0.9075	0.11

^(1)^ 7500 simulations.

^(2)^ On average.

^(3)^ On average calculated taking into consideration the RTE average.

Fifteen Scenarios (pre-management interventions). Concerning the input management, *P*_*RTE*⩵1_ varies in a [0.1447, 0.3113] range for workforce capacity health related (S9) and placement capacity (S13). The $\overset{-}{RTE}$ oscillates within [0.6128, 0.9357] for placement and workforce capacity (S15) and day care health related (S4). The (S4) shows the greatest probability (0.9260) of having an *RTE* score higher than 0.75, while the (S15) has the lowest one (0.3289) (Table 3, “Post-Interventions” column). From an output perspective, the mix placement capacity (S13) scenario has the highest *P*_*RTE*⩵1_ = 0.3445, while the lowest corresponds to workforce capacity health related (S9) *P*_*RTE*⩵1_ = 0.1616. The maximum $\overset{-}{RTE}=0.9311$ is reached by (S11), which combines residential and day care health related plus outpatient care. In contrast, (S6) outpatient care has the lowest $\overset{-}{RTE}=0.8408$. Concerning the probability of having an *RTE* higher than 0.75, (S6) shows the lowest value of 0.7464, while (S12) residential and health related placement capacity and outpatient availability has the highest at 0.9573 (Table 4, “Post-Interventions” column).

#### Stability and entropy of the system

**Fig 5:**
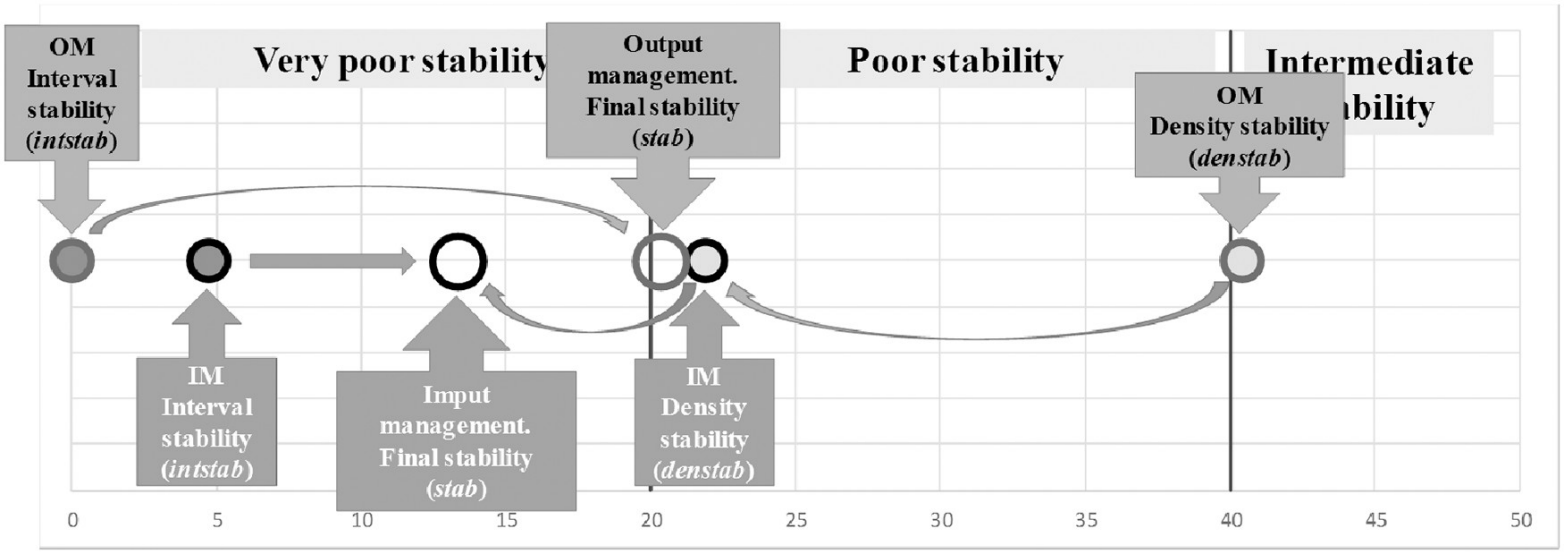
Global stability of the system: Initial situation *(stab =* 0.5 × *intstab +* 0.5 × *denstab)*.

Entire System (pre-management interventions). According to the input management, the interval stability (*intstab*) is very poor (small changes in data values can change *RTE* scores dramatically). Its density stability (*denstab*) is poor (small changes in data values can change *RTE* scores a lot). Finally, the stability of the entire system (*stab*), considering *w*_*int*_ = 0.5 and *w*_*den*_ = 0.5, is very poor (Fig 5 and Table 5, “pre” column).

**Table 5:** Scenario stability and entropy (in brackets the entropy in percentage up to its maximum).

		Input oriented results			Output oriented results		
Unit of analysis	Results	Pre	Post	Variation (%)	Pre	Post	Variation (%)
**Whole System**	Weighted Stability	13.34	13.46	0.90	20.23	20.20	-0.12
	Shannon´s entropy	3.83 (85.82%)	3.82 (85.60%)	-0.26	3.21 (72.01%)	3.21 (72.08%)	0.10
**S4**	Weighted Stability	24.08	24.11	0.10	19.46	19.43	-0.13
	Shannon´s entropy	2.37 (53.21%)	2.37 (53.06%)	-0.27	2.59 (58.11%)	2.59 (58.11%)	0.00
**S6**	Weighted Stability	11.22	10.91	-2.76	12.50	12.45	-0.40
	Shannon´s entropy	3.51 (78.61%)	3.50 (78.52%)	-0.11	3.25 (72.88%)	3.20 (71.86%)	-1.40
**S7**	Weighted Stability	9.54	10.53	10.38	22.98	22.99	0.07
	Shannon´s entropy	3.82 (85.60%)	3.73 (83.63%)	-2.30	2.63 (58.99%)	2.63 (59.02%)	0.05
**S8**	Weighted Stability	15.48	14.19	-8.37	23.61	23.51	-0.42
	Shannon´s entropy	3.32 (74.38%)	3.36 (75.30%)	1.23	2.70 (60.46%)	2.74 (61.44%)	1.61
**S9**	Weighted Stability	14.93	14.62	-2.11	18.48	18.38	-0.57
	Shannon´s entropy	3.24 (72.70%)	3.26 (73.01%)	0.43	3.18 (71.37%)	3.22 (72.25%)	1.23
**S10**	Weighted Stability	11.25	11.54	2.58	18.66	18.75	0.48
	Shannon´s entropy	3.70 (82.91%)	3.71 (83.14%)	0.29	3.16 (70.80%)	3.15	-0.22
**S11**	Weighted Stability	9.89	11.15	12.80	23.41	23.35	-0.26
	Shannon´s entropy	3.73 (83.56%)	3.69 (82.84%)	-0.86	2.80 (62.83%)	2.82 (63.34%)	0.82
**S12**	Weighted Stability	10.86	10.56	-2.72	23.21	23.15	-0.26
	Shannon´s entropy	3.75 (84.13%)	3.77 (84.43%)	0.36	2.58 (57.78%)	2.59 (58.04%)	0.45
**S13**	Weighted Stability	12.36	12.48	1.01	23.56	23.52	-0.19
	Shannon´s entropy	3.54 (79.35%)	3.50 (78.50%)	-1.07	2.66 (59.75%)	2.67 (59.79%)	0.08
**S14**	Weighted Stability	10.79	11.18	3.61	18.56	18.68	0.65
	Shannon´s entropy	3.64 (81.72%)	3.66 (82.09%)	0.45	3.17 (71.16%)	3.12 (70.00%)	-1.63
**S15**	Weighted Stability	5.87	5.89	0.43	20.04	19.98	-0.27
	Shannon´s entropy	4.11 (92.09%)	4.11 (92.25%)	0.17	3.17 (71.01%)	3.17 (71.05%)	0.06

The output management results show that the interval stability (*intstab*) is very poor, while the density stability (*denstab*) is intermediate (small changes in data values do not change *RTE* too much). The final stability (*stab*) is poor considering again that *w*_*int*_ = 0.5 and *w*_*den*_ = 0.5 (Fig 5 and Table 5, “pre” column).

According to the input management, the entropy of the global system is very high, 3.83 (85.82% up to the feasible maximum). In the output management analysis, it is also high, 3.21 (72.01% up to the feasible maximum). These results indicate that the Bizkaia MH system can be easily improved by selecting appropriate interventions (Table 5, “pre” column).

Fifteen Scenarios (pre-management interventions). From an input management perspective, the greatest *stab* = 24.08 corresponds to (S4) delivery of day care health related, while the lowest *stab* = 5.87 is for (S15) combination of placement capacity and workforce capacity for residential, day and outpatient care. In contrast, output management (S8) placement capacity health related care has the highest *stab* = 23.61, while (S6) outpatient care has the lowest *stab* = 12.50 (Table 5, “pre” column).

From an input management point of view, the Shannon’s entropy oscillates within [4.11 (92.09%), 2.37 (53.21%)], respectively, for (S15) places in the hospital and community and workforce capacity for both residential and outpatient care and (S4) day care health related. From an output management perspective, it varies within [2.58, 3.25] for (S12) hospital-community residential, day health related and outpatient care and (S6) outpatient care. Again, there are many opportunities to improve the MH System of Bizkaia (Table 5, “pre” column).

### RTE assessment: The impact of the mesomanagement interventions (“B”)

#### RTE results

Impact on the entire System (post-management interventions. Focusing our attention on input management, after the proposed three interventions, the $\overset{-}{RTE}$ increases slightly, and the difference (pre-post) is statistically significant (two-tailed *p* = 0.002, α = 0.05) while the variances cannot be considered equal (Levene´s test *p* < .001). *RTE* statistical distributions (pre and post) can be considered equal (Kolmogorov-Smirnov, α = 0.05 and 0.01; Table 3, “Post-Interventions” column). The output-oriented results indicate that $\overset{-}{RTE}$ decreases slightly, but the difference is not statistically significant (two-tailed *p* = 0.387, α = 0.05) and the variances can be considered equal (Levene´s test *p* = 0.356). Finally, the statistical distributions can be considered equal (Kolmogorov-Smirnov, α = 0.05 and 0.01; Table 4, “Post-Interventions” column).

The three management interventions cause a small but positive *RTE* increase in the service performance oriented to the input management. However, the situation from an output management perspective remains constant.

Impact on the fifteen scenarios (post-management interventions). Regarding the input management, the *P*_*RTE*⩵1_ increases in (S7), (S11), (S12) and (S13). The first one, placement capacity, shows the highest one (5.30%). However, in (S4), (S8), (S9), (S10), (S14) and (S15), *P*_*RTE*⩵1_ decreases, with (S15) places of hospital and community in addition to workforce capacity of outpatient care having the worst one (-2.63%). In (S6) outpatient care, *P*_*RTE*⩵1_ remains constant.

The $\overset{-}{RTE}$ increases in (S4), (S7), (S11), (S13), (S14) and (S15); in (S7) placement capacity of residential and day care, it increases to 2.34%. In (S10) workforce capacity total, $\overset{-}{RTE}$ remains constant, while in (S6), (S8), (S9) and (S12), it decreases, with (S12) placement capacity for residential care in the hospital and community plus day and outpatient care having the worst one.

Looking at the probability of having an *RTE* score higher than 0.75, it increases in all of the scenarios, except (S8), (S9), (S10) and (S12). (S14) shows the highest percentage (3.06%) and (S8) the worst after meso-interventions (-2.11%; Table 3).

Taking into account the output-oriented results, *P*_*RTE*⩵1_ is lower after the interventions in all of the scenarios, except (S6) outpatient care (3.49%) and (S13) placement capacity (2.51%). (S9) shows the highest negative impact (-5.08%).

$\overset{-}{RTE}$ increases in (S6) outpatient care and (S14) placement capacity and decreases in the remaining scenarios, with (S9) workforce capacity health related having the worst (-0.37%).

The probability of having an *RTE* score higher than 0.75 decreases in six of the eleven scenarios (S4, S7, S8, S9, S11 and S12), with (S12) being the most affected (-0.51%). In the remaining scenarios, it increases, and (S14) shows the highest percentage (0.66%; Table 4).

#### Stability and entropy of the system

Impact on the entire System (post-management interventions). In the input orientation and after the proposed interventions, the interval stability (*intstab*) remains constant, but the density stability (*denstab*) and *stab* have a slight increase (0.1%) that is not statistically significant. From an output management perspective, *intstab*, *denstab* and final stability (*stab*) remain constant (Table 5). The input-oriented results show that the entropy decreases (-0.26%), which is a positive impact. However, from an output management view, the entropy increases a bit (0.1%) (Table 5).

Impact on the fifteen scenarios (post-management interventions). The results oriented to input management show that (S11) places for hospital and community care and availability of outpatient care has the highest *stab* increase (12.80%), while (S8) places health related shows the highest decrease (-8.37%). However, output management analysis evidences that (S14) placement and workforce capacity for residential, day and outpatient care has the highest *stab* increase (0.65%), while (S8) reaches a relevant decrease (-0.42%; Table 5).

The input-oriented results show that the greatest negative variation (it increases) of the entropy corresponds to (S8) with 1.23%. However, in (S7) placement capacity, it decreases (positive impact) by -2.30%. Form an output orientation perspective, (S8) shows the highest increase (1.61%) again, while in (S14), the entropy significantly decreases (-1.63%; Table 5).

## Discussion

EDeS-MH allows decision makers to assess the *RTE* of the MH system for analysing the impact of potential management interventions and policies prior to their becoming real. This DSS is able to guide MH care managers and planners in designing evidenced-informed interventions as well as policies, reducing the risk associated with decision-making.

From a hybrid methodological approach, the Monte-Carlo simulation engine allows for the incorporation of the uncertainty of the real system into the DSS [22]. By using DEA models, both the resource use and the outcome production of SHA can be optimized. The utilization of the DESDE-LTC codification system [48] allows for the standardization, comparison and evaluation of MH systems, considering their main types of care provided and avoiding the terminology variability [44]. Finally, the fuzzy inference engine prototype integrates expert knowledge in the operational model [22].

In the initial situation of the entire Bizkaia MH system, the RTE indicators show that there is a relevant opportunity for improving the system performance from both input (resources) and output (outcomes) management points of view. On average, its RTE is 0.7831 and 0.8812, respectively, but it does not exactly mean that input management can be improved by 21.69% (maintaining constant the output production) or, otherwise, by 11.88% (maintaining constant the resource consumption). This is because of the inner variability of the system that is described by the proposed scenarios. In this case, the model implications reveal that planners and managers of the MH system of Bizkaia took into consideration the results obtained in the present study. They have approved the feasibility of the model for supporting decision-making processes and resource allocation.

The EDeS-MH allows decision makers to study the most likely specific consequences of interventions and policies on selected scenarios as well as to design new potential ones. This procedure is critical because, for example, a potentially neutral intervention at a global scale can be the result of a balance of negative and positive effects on real scenarios. By using this knowledge, decision makers can modify their proposals or design additional and corrective interventions on specific scenarios in a creative way, overcoming old ideas, e.g., for increasing the efficiency of a MH system, it is required to reduce the amount of resources used [55–57]. Expert knowledge (included in EDeS-MH) highly penalizes dramatic decreases in the inputs away from a specific minimum established by the B-MHCCM. Another old fashioned idea is that the efficiency of MH systems can be improved by increasing the level of outputs produced [55,58]. Again, expert knowledge identified non-standard outcomes where an uncontrolled increase away from another specific level–defined by the B-MHCCM- must also be penalized. The B-MHCCM understands these phenomena and can be used for a better understanding of the behaviour of complex MH systems under uncertainty.

From the perspective of the entire system, the proposed management interventions can be considered slightly positive (input management) and neutral (output management). Obviously, they imply very small changes in our real system (only a few professionals could be shifted and reassigned), so no dramatic effects were expected. However, the stability and Shannon’s entropy are very poor, so small changes in data values (due to an intervention or policy) can have a relevant impact, sometimes negative, in the efficiency of the entire system.

The analysis of the specific scenarios that are directly impacted by the management interventions showed that, in the input orientation, (S4) showed the highest $\overset{-}{RTE}$ and probability of having an *RTE* higher than 0.75 as well as a relative adequate stability and entropy. The performance of this scenario increased as a consequence of deinstitutionalization policies and the change in care provision from the hospital to the community, promoting the importance of day care [59,60].

The present findings support that it is possible to deliver an efficient day care in the community.

In (S7), (S11) and (S13), all of the indicators become better after the management interventions. These scenarios combine the placement capacity and availability of residential, day and outpatient care with utilization indicators (outcomes). For this reason, these decisions can be considered positive because the three main types of care in MH are combined in a balanced way.

From an output management perspective, (S11) showed the highest $\overset{-}{RTE}$, while (S12) has the highest probability of having an RTE higher than 0.75 as well as the best stability and entropy. These scenarios combine residential, day and outpatient care, so it is possible to provide an efficient delivery, supported by an appropriate balance between hospital and community care [61,62].

However, (S6) outpatient care showed an improved situation after the mesomanagement interventions. In addition, (S14) evidenced the better stability and entropy improvements. Therefore, after carrying out the interventions, the provision of outpatient care improved.

Currently, the EDeS-MH has some drawbacks related to the difficulty of modelling interventions/policies in complex and interrelated MH systems under conditions of uncertainty. Decisions have to be translated into data variations (usually in the input variables) and expectations (usually in the output variables). For a specific intervention, decision makers can easily identify first level variations for the inputs (the variables that are directly impacted by the intervention; for this paper: the workforce variations due to the reassign process), but they have difficulties in identifying and assessing the expected consequences in both the outcomes and second, third, etc., level variables–inputs or outputs- (that modify their values as a cause of the primary changes, for example, data variations due to the hospital structural changes) according to a causal model. The EbCA model [50] is used to guide this process.

There were some limitations in our study. The analysis did not included 65 beds for long-term care financed by the Department of health of Bizkaia but placed in another province. These beds where part of a nested system within the MH system of Gipuzkoa so it was agreed with the experts from the public agency to exclude this facility from the counting of service availability and placement capacity in the Bizkaia MH care system. Bizkaia is a relative small province in the Spanish MH system with a centralised management and, due to that, the procedure have to be repeated for other provinces or regions in order to increase the variability. In the RTE assessment the majority of the statistical distributions were triangular (see section “Randomization of the original data” for a better understanding of their structure), this distribution can be considered very appropriate for this case but other (trapezoidal, uniform, etc.) can be also used, also with different weights. The expert-based ranges for data values interpretation (according to the B-MHCCM) as well as other parameters can be modified in order to check the DSS sensitivity to these changes. The DSS cannot yet interpret complex relationships between variables in day and outpatient care but it is ready to manage causal relationships when residential care variables are considered the “effect” of day and outpatient provision. All the variables as well as D (day care) and O (outpatient care) constructs are analysed taking into consideration only 5 fuzzy sets. In order to compare RTE statistical distributions, additional non-parametric tests can be also included in the DSS. In order to analyse the viability of the DSS, only the impact of meso-management interventions was assessed; macro-management interventions (or complex policies) can also be taken in consideration for complex viability analysis.

In conclusion, both the EDeS-MH and the procedure followed work properly and allow decision and policy makers to analyse and propose new alternatives that will have an impact on a MH system of selected interventions and policies prior to their becoming real.

## Conclusions

Decision makers reported that the EDeS-MH is useful for helping them to (i) structure their minds in designing new interventions (i.e., to describe real problems, to: identify critical variables that are directly concerned, to identify secondary variables involved by using causal reasoning and, finally, to assess their expected outcomes–variables and amounts); (ii) understand the behaviour of the global system as well as of the corresponding SHAs in specific scenarios; (iii) identify critical scenarios that could have a negative reaction; (iv) identify post-intervention improvements in specific areas and scenarios; and, finally, (v) design nested-interventions (policies) from a long-term perspective.
